# Incidence and risk factors of suicide among patients with pancreatic cancer: A population-based analysis from 2000 to 2018

**DOI:** 10.3389/fonc.2022.972908

**Published:** 2022-08-19

**Authors:** Yifei Ma, Jun Lyu, Bao Yang, Tianao Yan, Qingyong Ma, Zheng Wu, Zheng Wang, Hairong He

**Affiliations:** ^1^ Department of Hepatobiliary Surgery, The First Affiliated Hospital of Xi’an Jiaotong University, Xi’an, China; ^2^ Department of Surgical Intensive Care Unit, The First Affiliated Hospital of Xi’an Jiaotong University, Xi’an, China; ^3^ Department of Clinical Research, The First Affiliated Hospital of Jinan University, Guangzhou, China; ^4^ Department of Clinical Research Center, The First Affiliated Hospital of Xi’an Jiaotong University, Xi’an, China

**Keywords:** pancreatic cancer, the SEER database, SMRs, risk factors, suicide

## Abstract

**Background:**

The rate of suicide within one year after diagnosis in pancreatic cancer patients are high, but suicide studies based on the current large-scale data are still a vacancy. Our study aimed to determine, compared to the general population, the standardized mortality ratios (SMRs) of suicide and risk factors associated with pancreatic cancer patients committing suicide to provide clues for prevention.

**Methods:**

We collected 199,604 patients diagnosed with pancreatic cancer between 2000 and 2018 from the SEER database. Multivariate logistic regression and multivariate Cox regression were applied to determine the risk factors independently affecting the suicide outcome of pancreatic cancer patients.

**Results:**

A total of 180 suicide deaths were observed in the cohort, yielding an overall suicide rate of 88.05 per 100,000 person-years and an SMR of 6.43. In multivariate analyses, males (HR: 12.798, 95% CI: 7.471-21.923), unmarried (HR: 1.826, 95% CI: 1.205-2.767), and divorced, separated or widowed (HR: 1.779, 95% CI: 1.230-2.572) were found associated with a higher risk of suicide. While race black (HR: 0.250, 95% CI: 0.110-0.567), diagnosed with pancreatic neuroendocrine tumor (HR: 0.487, 95% CI: 0.276-0.859), received chemotherapy (HR: 0.456, 95% CI: 0.323-0.646), and received surgical procedures (HR: 0.553, 95% CI: 0.342-0.895) were indicated might protective factors.

**Conclusions:**

The 199,604 pancreatic cancer patients diagnosed between 2000 and 2018 had an overall suicide rate of 88.05 per 100,000 person-years and an SMR of 6.43 compared to the U.S. general population. Male, white, unmarried, and diagnosed with pancreatic adenocarcinoma patients were associated with a higher risk of suicide, while cancer-directed surgery and chemotherapy might indicate protective factors. The screening and prevention process should be enhanced for pancreatic cancer patients with adverse risk factors. Moreover, it is reasonable to assume that timely cancer-directed treatment might help reduce the subsequent suicide risk of pancreatic cancer patients.

## Highlights

- A total of 180 suicide deaths were observed in 199,604 pancreatic cancer patients from 2000 to 2018, representing 0.10% of all death records, yielding an overall suicide rate of 88.05 per 100,000 person-years.
- As chemotherapy and cancer-directed surgery indicated protective factors, it is reasonable to assume that timely cancer-directed treatment might help reduce the subsequent suicide risk of pancreatic cancer patients.

## Introduction

Suicide is the culmination of unmanaged negative emotions (1), posing a severe health burden to society. In 2020, suicide was the second leading cause of death in the 10-34 age group and 12th among all age groups in the United States (2). The 2020 U.S. overall suicide rate was 13.5 per 100,000 standard population, resulting in 45,979 deaths, yielding an increase of 30% over the past 20 years (2). As reported, approximately 70% of suicides in patients over 60-year-old were illness-related (3, 4). Cancer is a major health problem worldwide and the second leading cause of death in the United States (5). Its diagnosis and long treatment process often cause not only physical or financial burdens but also severe psychological stress to patients (6, 7), which has been demonstrated to double the risk of suicide compared to the general population (3, 8–10). However, these additional life losses are potentially preventable with early and appropriate psychological intervention to some extent (11). The Cancer Taskforce in the U.K. further highlighted that better management of depression could also improve cancer patients’ outcomes (11). Therefore, it is proved necessary and desperate to identify patient groups with a higher risk of suicide at the population level and conduct timely psychological screening or intervention measures (1).

Pancreatic cancer is the fourth leading cause of cancer death in the United States, with a five-year survival rate of only 11% (5). Several surveys have elaborated on the significant clinical association between pancreatic cancer and depression, which might be more robust than other advanced abdominal malignancies (12–14). As reported, there was a 38-45% depression rate among pancreatic cancer patients and the highest suicide rate within a year after diagnosis of all cancers (15–17). However, suicide studies focusing on pancreatic cancer are scarce, and there are no reliable conclusions based on a large, contemporary population. Therefore, based on the SEER database, the objective of our study is to determine the suicide rate of pancreatic cancer patients from 2000 to 2018 in the U.S., compared with the general population in different demographic and clinicopathological subgroups, identify factors that potentially increase the suicide risk, and provide research evidence for reducing the risk of suicide in patients with pancreatic cancer.

## Materials and methods

### Data source and selection

**Figure 1:**
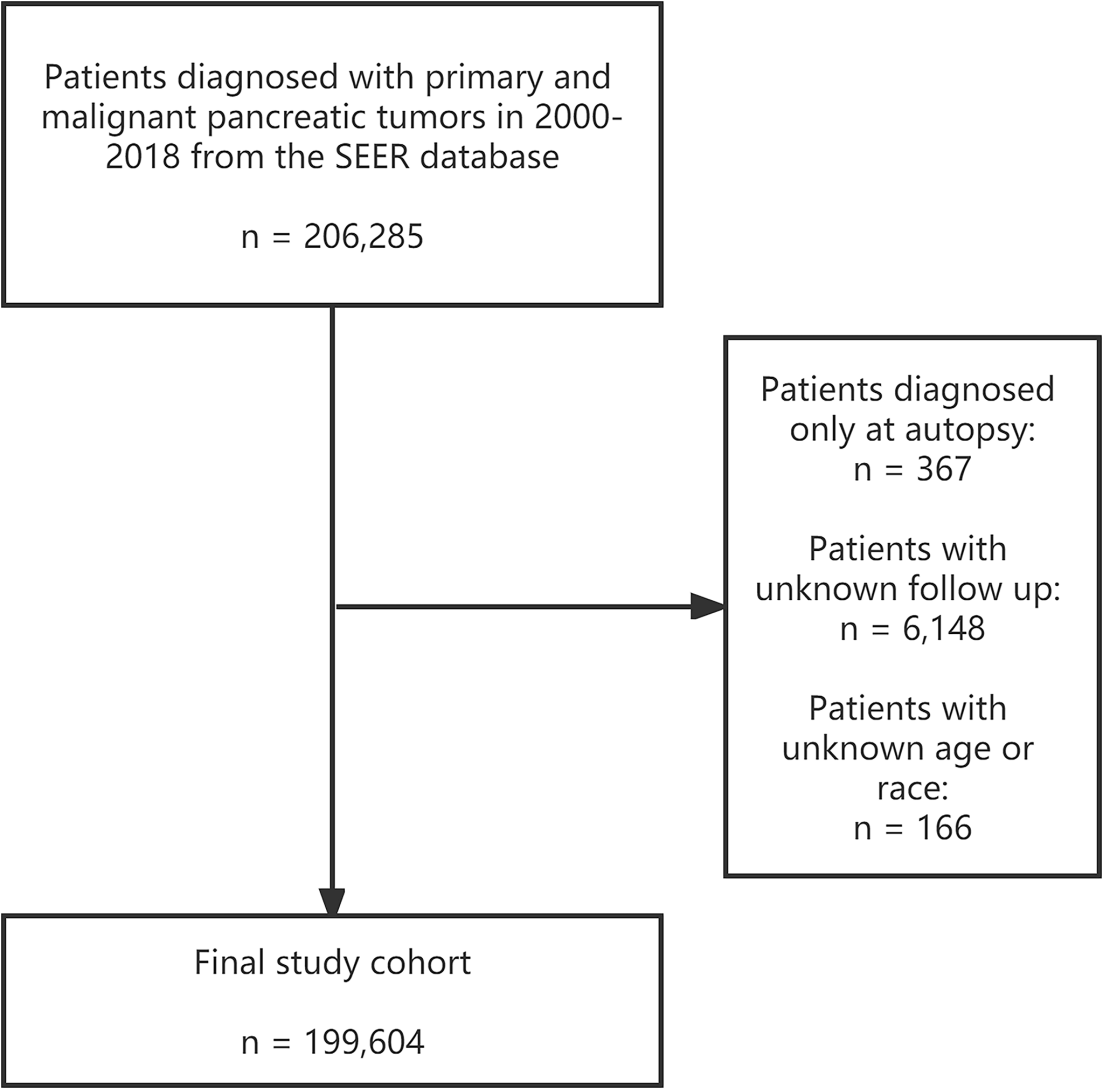
Screening process of 199,604 patients diagnosed with primary and malignant pancreatic tumors in 2000-2018 in the SEER database. SEER: Surveillance, Epidemiology, and End Results program.

We extracted patients pathologically diagnosed with pancreatic cancer between 2000 to 2018 from the National Cancer Institute’s Surveillance, Epidemiology, and End Results (SEER) program. The subdatabase “Incidence - SEER Research Plus Data, 18 Registries, Nov 2020 Sub (2000-2018)” was selected as the resource for our study population. In addition, the U.S. general population data were also accessed from the National Vital Statistics Reports by National Center for Health Statistics for comparison (18). Patients with primary pancreatic tumors with malignant behavior from 2000 to 2018 were identified by the primary site codes C25.0-C25.9 in ICD-O-3 (19). Patients with unknown age, race, follow-up time, or only autopsy diagnosis were excluded from the study cohort. A total of 199,604 pancreatic cancer patients who met the study requirements were finally enrolled. The detailed screening process is shown in **Figure 1**. The primary outcome of concern was suicide deaths, with the cause of death described as “Suicide and Self-Inflicted Injury” in the SEER database with the International Classification of Diseases, Tenth Revision (ICD-10) codes U03, X60-X84, and Y87.0. And definitions of these codes by The World Health Organization (WHO) can be found on this website: https://icd.who.int/browse10/2019/en. The software of SEER*Stat (version 8.3.9) was used to extract the patients.

### Variables identified

The following patients’ characteristics of interest were all enrolled: gender, race (white, black, others), state, age of diagnosis, year of diagnosis, marital status, primary tumor site (head, body or tails, others), histological type, histological grade, SEER summary stage (localized, regional, distant), radiotherapy records (yes, no), chemotherapy records (yes, no), cancer-directed surgical procedures (yes, no), and survival months after diagnosis.

Among them, age of diagnosis was the only continuous variable that could be included in the multivariate analysis as a potential risk factor for suicide. To make the results more intuitive, we obtained the optimal cut-off value through X-tile software and divided the patients into < 53 years old, 54-67 years old, and ≥ 68 years old groups (20). Marital status was classified as married, unmarried, divorced, separated, or widowed (DSW). The histological types were classified as pancreatic ductal adenocarcinoma (PDAC), pancreatic neuroendocrine tumor (pNET), pancreatic cystic neoplasm (PCN), and unknown/other types (21). If the patient’s survival months were 0, it was approximated to 0.5 for analysis convenience (22). In addition, patients were classified as < 2 months, 2-12 months, and ≥ 13 months based on the number of follow-up months since diagnosis. The cut-off value of two months was chosen as the best estimate of the reasonable window between diagnosis and initiation of treatment (22, 23).

### Statistical analysis

Suicide rates were calculated from the number of suicides reported per 100,000 person-years follow-up time. Chi-square tests were performed to determine whether differences in rates between groups, or linear trends between differences, were statistically significant. Moreover, the Bonferroni-corrected P-value was applied for multiple comparisons when necessary.

Standardized mortality ratios (SMRs) were the ratio of observed suicides to expected suicides, as defined. And the number of expected suicides was the product of the suicide rate in the general population and the person-years of follow-up in the study subgroup. The suicide rates of the general U.S. population were adjusted by year, age, gender, and race, based on statistics from the National Center for Health Statistics (1, 18). Moreover, the 95% confidence intervals (CI) of SMRs were figured out by mid-P tests, which were achieved by treating the number of observed deaths as variables satisfying Poisson distributions (24).

Multivariate analyses were applied to determine the risk factors independently affecting the suicide outcome of pancreatic cancer patients. Multivariate logistic regression was performed based on patients’ observed number of suicide deaths (1). We also established a multivariate Cox regression model to make the results more accurate, considering that the survival and non-suicide deaths were censored (1). The following variables were included in both analyses: age of diagnosis, year of diagnosis, sex, race, marital status, primary tumor site, histological type, histological grade, stage, radiotherapy records, chemotherapy records, and cancer-directed surgery records. The results of the multivariate analysis were represented by odds ratios (OR) and hazard ratios (HR), with their 95% CI. All statistical tests were two-sided, and *P* < 0.05 indicated statistical significance. Rstudio software (version 3.6.3) and SPSS (version 26.0) were adopted for all statistical analyses in our study.

## Results

### Patients baseline characteristics

In general, there were 199,604 patients diagnosed with pancreatic cancer from 2000 to 2018 in our study cohort, and the entire follow-up time was 204,437.58 person-years. By November 2020, 176,627 (88.49%) had been confirmed dead, and 180 patients died of suicide, yielding a total suicide rate of 0.09%. In the whole pancreatic cancer patients cohort, the mean age of diagnosis was 69.7; 50.5% of them were male, and the white race was the predominant race (80.2%). 53.1% of patients were married, 29.8% were once married (divorced, separated, or widowed, DSW), and 12.7% never married. As for clinicopathologic features, 51.2% of patients had a distant stage when diagnosed, and 47.6% of tumors were found in the pancreatic head. More than half of the histologic types of tumors are PDAC (67.1%). Overall, 13.4% of patients received radiotherapy, 43.3% received chemotherapy, and 18.0% underwent cancer-directed surgery.

Of the 180 patients who committed suicide, The vast majority were male (91.6%) and white (90.5%). 96 (53.3%) patients were married, and 44 (24.4%) were previously married. In addition, 89 (49.4%) patients had a distant stage, 89 tumors were found in the pancreas head, and 68.9% were PDAC. 17 (9.4%) patients received radiotherapy, 68 (37.7%) received chemotherapy, and 37 (20.5%) patients had surgical procedures. Furthermore, 77.2% (139) of suicides occurred within one year of diagnosis of pancreatic cancer, with 45.5% (82) occurring within two months. More details are presented in **Table 1**.

**Table 1:** Baseline characteristics, suicide rates, and standardized mortality ratios (SMR) among pancreatic cancer patients (2000-2018).

Characteristics	Total (%)	Suicides (%)	Person-years	Suicides per 100,000 Person-years	*P*	SMR	95% CI
Total	199,604 (100.0)	180 (100.0)	204,437.58	88.05		6.43	5.49 - 7.37
Year of diagnosis					0.059$		
2000-2005	51,047 (25.5)	49 (27.2)	55,323.79	88.57		7.03	5.16 - 9.26
2006-2012	73,102 (36.6)	56 (31.1)	86,121.00	65.02		4.75	3.50 - 5.99
2013-2018	75,455 (37.8)	75 (41.6)	62,992.79	119.06		7.73	5.98 - 9.48
Age groups					< 0.001$		
≤ 53	20,966 (10.5)	19 (10.6)	41,090.71	46.24		2.36	1.43 - 3.68
54-67	61,894 (31.0)	59 (32.8)	76,619.67	77.00		4.40	3.28 - 5.52
68+	116,744 (58.4)	102 (56.7)	86,727.21	117.61		7.49	6.04 - 8.94
Sex					< 0.001		
Female	98,690 (49.4)	15 (8.3)	101,974.21	14.71		2.37	1.33 - 3.94
Male	100,914 (50.5)	165 (91.6)	102,463.38	161.03		7.06	5.98 - 8.13
Race					0.001#		
White	160,190 (80.2)	163 (90.5)	164,759.79	98.93		5.47	4.63 - 6.31
Black	23,959 (12.0)	6 (3.3)	22,743.33	26.38		3.61	1.29 - 7.71
Other	15,455 (7.7)	11 (6.1)	16,934.46	64.96		9.69	4.91 - 17.91
Marital status					0.200		
Married	106,150 (53.1)	96 (53.3)	124,000.17	77.42		5.65	4.52 - 6.78
Unmarried	25,531 (12.7)	31 (17.2)	26,736.50	115.95		8.46	5.68 - 11.89
DSW	59,516 (29.8)	44 (24.4)	44,565.38	98.73		7.21	5.25 - 9.67
Unknown	8,407 (4.2)	9 (5.0)	9,135.54	98.52		7.19	3.08 - 13.15
Stage					< 0.001$		
Localized	21,505 (10.7)	27 (15.0)	45,751.88	59.01		4.31	2.83 - 6.22
Regional	57,185 (28.6)	50 (27.7)	85,319.67	58.60		4.28	3.16 - 5.63
Distant	102,280 (51.2)	89 (49.4)	58,263.21	152.76		11.15	8.81 - 13.44
Unknown/unstaged	18,634 (9.3)	14 (7.7)	15,102.83	92.70		6.77	3.67 - 11.19
Grade					0.895#		
Grade I/II	36,633 (18.3)	44 (24.4)	74,928.00	58.72		4.29	3.11 - 5.73
Grade III/IV	26,472 (13.2)	15 (8.3)	26,573.46	56.45		4.12	2.33 - 6.89
Unknown	136,499 (68.3)	121 (67.2)	102,936.13	117.55		8.58	7.05 - 10.11
Primary site					0.380		
Head	95,063 (47.6)	89 (49.4)	104,953.88	84.80		6.19	4.90 - 7.46
body or tails	49,421 (24.7)	44 (24.4)	54,700.96	80.44		5.87	4.27 - 7.87
Others	55,120 (27.6)	47 (26.1)	44,782.75	104.95		7.66	5.66 - 10.25
Histological type					< 0.001#		
PDAC	134,100 (67.1)	124 (68.9)	116,946.29	106.03		7.75	6.38 - 9.10
pNET	11,538 (5.7)	18 (10.0)	41,358.83	43.52		3.16	1.89 - 5.01
PCN	7,752 (3.8)	9 (5.0)	12,991.96	69.27		5.00	2.25 - 9.61
Unknown/Other types	46,214 (23.1)	29 (16.1)	33,140.50	87.51		6.44	4.27 - 9.16
Radiotherapy					< 0.001		
No/Unknown	172,819 (86.5)	163 (90.5)	160,203.13	101.75		7.43	6.27 - 8.55
Yes	26,785 (13.4)	17 (9.4)	44,234.46	38.43		2.81	1.62 - 4.46
Chemotherapy					< 0.001		
No/Unknown	112,980 (56.6)	112 (62.2)	96,603.58	115.94		8.46	6.91 - 10.06
Yes	86,624 (43.3)	68 (37.7)	107,834.00	63.06		4.60	3.50 - 5.69
Surgery					< 0.001		
No/Unknown	163,655 (81.9)	143 (79.4)	105,914.25	135.01		9.86	8.25 - 11.48
Yes	35,949 (18.0)	37 (20.5)	98,523.33	37.55		2.74	1.93 - 3.78
Months elapsed from diagnosis					< 0.001$		
≤ 2	76,951 (38.5)	82 (45.5)	6,607.42	1241.03		90.59	71.39 - 110.83
3-12	73,512 (36.8)	57 (31.6)	39,355.17	144.83		10.57	7.82 - 13.30
13+	49,141 (24.6)	41 (22.7)	158,475.00	25.87		1.89	1.35 - 2.56

SMR, Standardized mortality ratio. CI, 95% confidence interval. PDAC, pancreatic ductal adenocarcinoma; pNET, pancreatic neuroendocrine tumor; PCN, pancreatic cystic neoplasm. $ The chi-square test for linear trend was used for ordinal multi-categorical variables. # The Bonferroni-corrected P-value was used for multiple comparisons.

### The difference in suicide rates and SMRs

**Figure 2:**
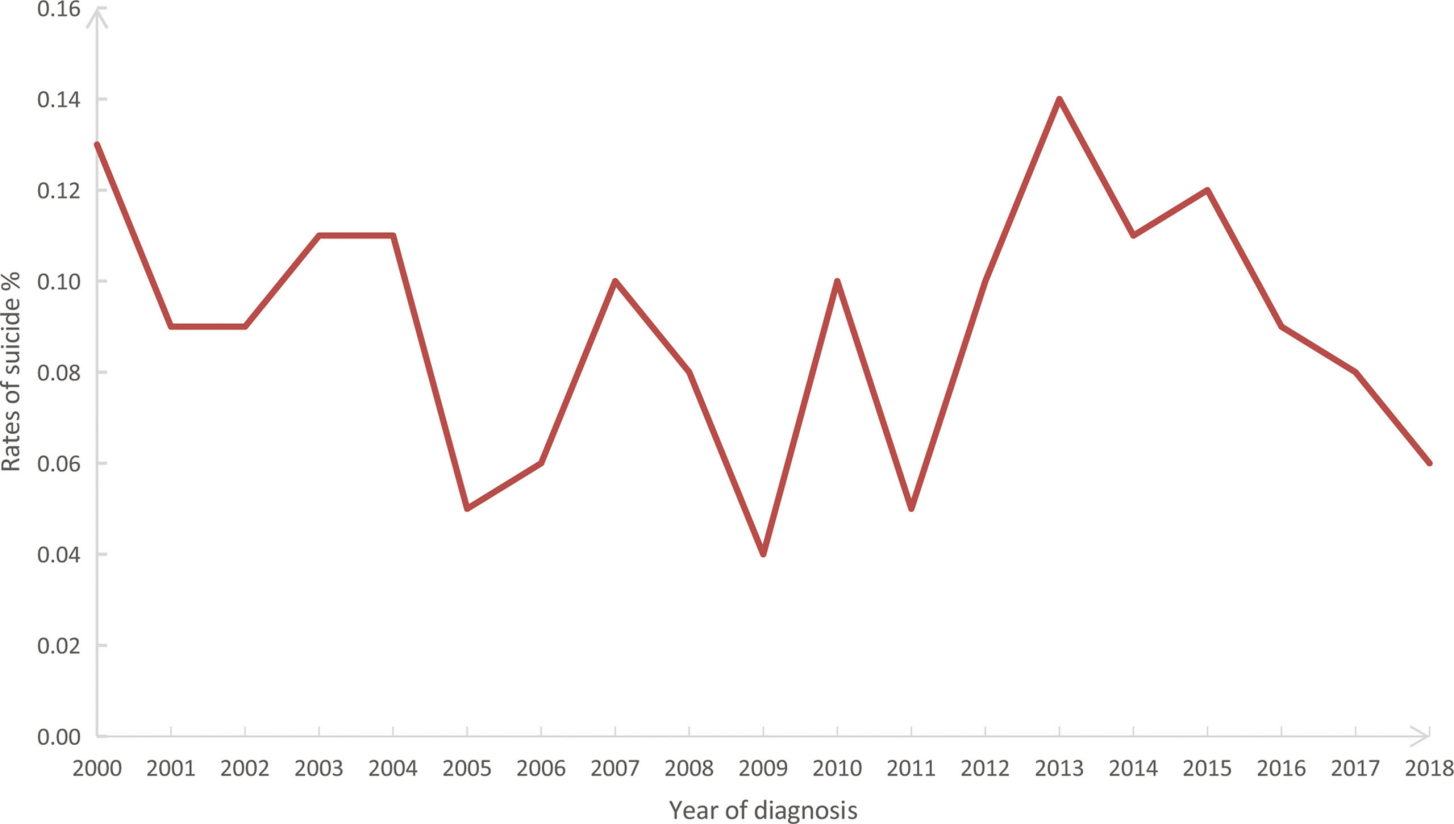
Annual suicide rates (%) from 2000 to 2018 among 199,604 patients diagnosed with pancreatic cancer in the SEER database. SEER: Surveillance, Epidemiology, and End Results program.

**Figure 3:**
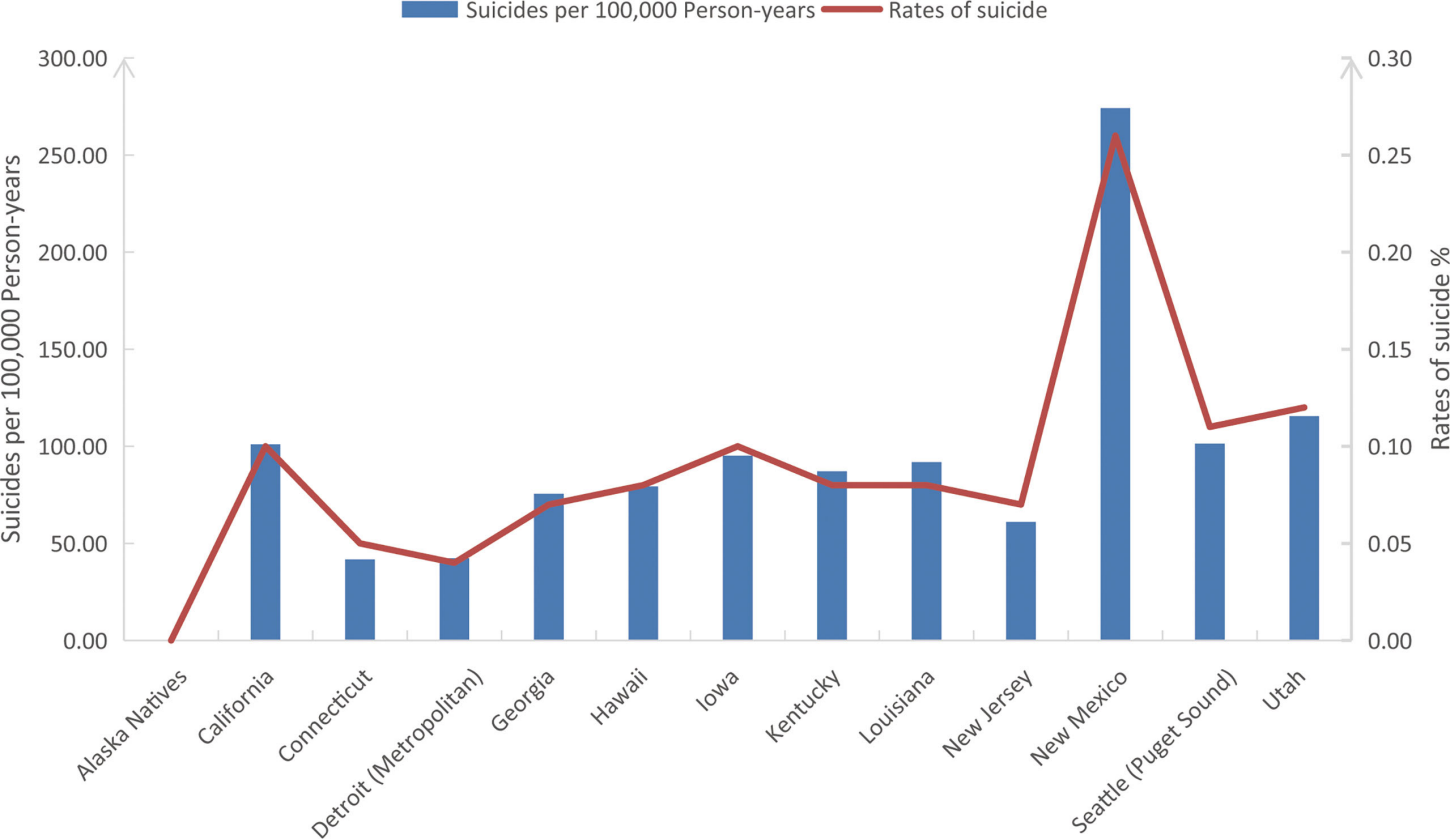
The overall suicide rates (%) and suicides per 100,000 person-years of pancreatic cancer patients in all SEER registered states from 2000 to 2018. SEER: Surveillance, Epidemiology, and End Results program.

In our study cohort from SEER, The overall suicide rate among pancreatic cancer patients from 2000 to 2018 was 88.05 per 100,000 person-years, compared with a national average for the 65-74 age group of 13.70 during the same period. Rates of suicide each year are shown in **Figure 2**, with the highest in 2013 (0.14%) and the lowest in 2009 (0.04%). However, chi-square tests observed no significant linear trend for suicide rates in pancreatic cancer patients over the following period (*P* = 0.828). In addition, the overall suicide rate for pancreatic cancer patients in each state registered in the SEER database from 2000 to 2018 is presented in **Figure 3**, where patients in New Mexico were observed with the highest suicide rate (274.17 per 100,000 person-years).

In subgroup comparisons of suicide rates among pancreatic cancer patients, higher suicide rates were observed in males (*P* < 0.001), whites (*P* = 0.001), patients diagnosed with PDAC (*P* < 0.001), and patients without clinical treatment (without radiotherapy, chemotherapy, or surgery, *P* < 0.001). The suicide rate increased with the growing age and tumor stage while decreasing with the extension of follow-up time, all showing statistically significant linear trends (*P* < 0.001). However, there were no significant differences in suicide rates among patients by year of diagnosis, marital status, histological grade, and primary tumor site. Pancreatic cancer patients in the study cohort had an SMR of 6.43 (95% CI: 5.49-7.37) for suicide, compared with the U.S. general population aged 65-74 during the same period, with 7.06 (95% CI: 5.98-8.13) for males, 5.47 (95% CI: 4.63-6.31) for whites, and 8.46 (95% CI: 5.68-11.89) for unmarried patients. More details are shown in **Table 1**.

### Risk factors associated with suicide

Through multivariate logistic regressions, we found that the risk of suicide was 11.98 times higher in males than females (95% CI: 7.000-20.513) in our study population. Black patients had a lower risk of suicide than whites (OR: 0.241, 95% CI: 0.106-0.548). Unmarried (OR: 1.703, 95% CI: 1.123-2.584) or DSW (OR: 1.570, 95% CI: 1.085-2.270) patients were more likely to commit suicide than married patients.

In multivariate Cox regressions, males (HR: 12.798, 95% CI: 7.471-21.923), unmarried (HR: 1.826, 95% CI: 1.205-2.767), and DSW (HR: 1.779, 95% CI: 1.230-2.572) were also found associated with a higher risk of suicide. While race black (HR: 0.250, 95% CI: 0.110-0.567), diagnosed with pNET (HR: 0.487, 95% CI: 0.276-0.859), received chemotherapy (HR: 0.456, 95% CI: 0.323-0.646), and received surgical procedures (HR: 0.553, 95% CI: 0.342-0.895) were indicated might protective factors. Nevertheless, the year or age of diagnosis, tumor stage, grade, primary site, or received radiotherapy or not had no statistically significant association with suicide risk. All the detailed multivariate results are shown in **Table 2**.

**Table 2:** Odds ratios and hazard ratios of suicide among pancreatic cancer patients (2000-2018) by multivariate analyses.

Characteristics	Logistic regression model			Cox proportional hazards model		
	OR	95% CI	*P*-value	HR	95% CI	*P*-value
Year of diagnosis						
2000-2005	1	—	—	1	—	—
2006-2012	0.753	0.512 - 1.109	0.152	0.763	0.516 - 1.128	0.175
2013-2018	0.901	0.619 - 1.310	0.585	1.127	0.770 - 1.650	0.539
Age groups						
≤ 53	1	—	—	1	—	—
54-67	1.125	0.666 - 1.901	0.661	1.300	0.769 - 2.197	0.328
68+	1.162	0.694 - 1.946	0.568	1.578	0.944 - 2.639	0.082
Sex						
Female	1	—	—	1	—	—
Male	11.988	7.000 - 20.531	< 0.001	12.798	7.471 - 21.923	< 0.001
Race						
White	1	—	—	1	—	—
Black	0.241	0.106 - 0.548	0.001	0.250	0.110 - 0.567	0.001
Other	0.751	0.407 - 1.384	0.358	0.737	0.400 - 1.358	0.327
Marital status						
Married	1	—	—	1	—	—
Unmarried	1.703	1.123 - 2.584	0.012	1.826	1.205 - 2.767	0.005
DSW	1.570	1.085 - 2.270	0.017	1.779	1.230 - 2.572	0.002
Unknown	1.462	0.735 - 2.909	0.279	1.404	0.705 - 2.792	0.334
Stage						
Localized	1	—	—	1	—	—
Regional	0.781	0.474 - 1.284	0.329	0.953	0.582 - 1.560	0.849
Distant	0.747	0.462 - 1.209	0.236	1.367	0.844 - 2.213	0.203
Unknown/unstaged	0.752	0.372 - 1.522	0.429	0.814	0.406 - 1.632	0.562
Grade						
Grade I/II	1	—	—	1	—	—
Grade III/IV	0.515	0.281 - 0.942	0.031	0.637	0.348 - 1.163	0.142
Unknown	0.860	0.558 - 1.325	0.493	0.903	0.593 - 1.373	0.632
Primary site						
Head	1	—	—	1	—	—
body or tails	0.866	0.592 - 1.267	0.460	0.916	0.628 - 1.335	0.648
Others	0.920	0.634 - 1.337	0.663	0.995	0.687 - 1.441	0.979
Histological type						
PDAC	1	—	—	1	—	—
pNET	1.249	0.705 - 2.213	0.446	0.487	0.276 - 0.859	0.013
PCN	1.258	0.635 - 2.493	0.510	0.941	0.472 - 1.876	0.863
Unknown/Other types	0.627	0.402 - 0.977	0.039	0.628	0.403 - 0.979	0.040
Radiotherapy						
No/Unknown	1	—	—	1	—	—
Yes	0.689	0.401 - 1.186	0.179	0.638	0.373 - 1.092	0.101
Chemotherapy						
No/Unknown	1	—	—	1	—	—
Yes	0.804	0.572 - 1.130	0.210	0.456	0.323 - 0.646	< 0.001
Surgery						
No/Unknown	1	—	—	1	—	—
Yes	1.045	0.644 - 1.695	0.858	0.553	0.342 - 0.895	0.016

OR, Odds ratio; HR, Hazard ratio; CI, 95% confidence interval. PDAC, pancreatic ductal adenocarcinoma; pNET, pancreatic neuroendocrine tumor; PCN, pancreatic cystic neoplasm.

## Discussion

Several large population-based studies have previously reported that cancer patients had a significantly higher risk of suicide than the general population (1, 11, 25–27), with one recent study suggesting 4.4 times (1). The decision mechanism of suicide is complex and influenced by multiple physiological, psychological, and social factors (11), and its occurrence is often abrupt and impulsive. So predicting suicidal ideations might be difficult (28). However, appropriate needs-based psychological interventions could still effectively reduce the incidence of adverse outcomes (1, 11). Previous surveys have also shown a high rate of depression and suicide in pancreatic cancer patients (16, 29). Therefore, our findings are expected to provide some hints or inspiration about suicide prevention in patients with pancreatic cancer for psychological and clinical workers.

The reason for the high suicide rate among pancreatic cancer patients is a complex issue. The prognosis of pancreatic cancer patients is abysmal, and the effective treatment is still relatively single (30). The insidious onset and early metastasis of pancreatic adenocarcinoma also reduce the surgical resection rate. The high incidence of depression caused by these characteristics may be the root cause of the high suicide rate of pancreatic cancer (29). Feelings of doom at diagnosis, complications from advanced cancer, chronic pain, and the financial strain of treatment might also play a role (31). In general, our study obtained an SMR of 6.43 (95% CI: 5.49-7.37) in pancreatic cancer patients compared with the general U.S. population aged 65-74. Previous studies have already concentrated on suicide death among patients with other gastrointestinal cancers, and the SMRs were 2.26 (95% CI: 1.78-2.84) for liver cancer (23), 4.07 (95% CI: 3.18-5.13) for gastric cancer (32), and 5.45 (95% CI: 4.66-6.35) for esophageal cancer (22). Unsurprisingly, pancreatic cancer patients have been reported to be the most depressed among gastrointestinal tumors (13, 14), which corroborates our findings. In addition, a formal study focusing on pancreatic cancer patients between 1995 and 2005 concluded that the rate of pancreatic cancer suicide was 135.4 cases per 100,000 person-years, with an SMR of 10.8 (12). Although we did not find a downward trend in suicide rates among pancreatic cancer patients between 2000 and 2018, the problem of patient suicide would eventually improve as research progresses, as compared.

In terms of differences in suicide rates among pancreatic cancer patients across SEER registered states, we found that compared with the overall pancreatic cancer cohort, New Mexico has a higher proportion of white patients (91.3% vs. 80.2%, *P* < 0.001) but a significantly lower proportion of patients undergoing chemotherapy (35.6% vs. 43.3%, *P* < 0.001) and surgery (13.9% vs. 18.0%, *P* < 0.001). This finding might partly explain why New Mexico has a significantly higher suicide rate among pancreatic cancer patients than other states and may also confirm our findings and inference that cancer treatment is associated with a lower risk of suicide among pancreatic cancer patients.

Several studies have demonstrated an association between age and suicide rates in cancer patients (1, 11). Overall, younger cancer patients were observed with a higher SMR, while the older patients had a higher risk of suicide (1). In our study, although we found the suicide rate significantly increased with patients’ age, it was not associated with suicide risk in the multivariate analysis, whether age was included as a continuous variable or a categorical variable. The results might be related to the older diagnostic age of pancreatic cancer patients, with an overall average age of 70. Regarding gender differences, our results are consistent with the previous studies (1, 22, 23). Among pancreatic cancer patients, males were 12 times more likely to commit suicide than females, with an SMR of 7.06. Males also have a higher risk of suicide in the general population (33), and the difference is magnified among cancer patients. This phenomenon is often explained by the fact that although females have a higher incidence of psychological disorders (34), males tend to have more direct means of ending their lives and a greater ability to act out their momentary impulses (35). For example, in the United States, the proportion of firearm-related suicides among males far exceeded that of females (2). In addition, although males might face more intractable sources of stress (22), they are less likely to have psychological problems diagnosed (34). The unmanaged stress could finally turn into a vicious ending. Our results were also supported by several studies examining the effect of race or marital status on suicide risk in cancer patients (22, 23, 32). Black pancreatic cancer patients have only a quarter of the risk of suicide compared with whites, which some studies suggest might be related to religion or cultural factors (36). Some researchers also thought it could be explained that depression symptoms are less strongly associated with hopelessness in blacks than in whites (37). Unmarried or DSW status patients had more potent suicide ideation than married patients. A large study has highlighted the importance of social support in preventing suicide among cancer patients (17). With the help of a trusted partner, cancer patients could better understand their disease and prognosis, and their financial burden could also be reduced.

In terms of the impact of the clinicopathological features of pancreatic cancer on patient suicide, we found that patients with pancreatic adenocarcinoma might suffer a higher risk of suicide, approximately twice that of patients with pNET. Pancreatic adenocarcinoma has been reported to have the highest degree of malignancy, the worst prognosis, and a low excision rate in pancreatic cancer (21). Studies have implied that it is difficult for patients to actively cope with the notorious diagnosis of pancreatic cancer, which increases the risk of passive coping mechanisms, leading to a poor prognosis (38). In addition, our study found no association between pancreatic cancer stage, histological grade, or primary tumor site and suicidal tendencies. The onset of pancreatic cancer is insidious, and the early symptoms are often not obvious (30). The distant metastasis often occurs when detected. The latest statistics showed that 30-35% of pancreatic cancer patients present locally advanced, and 50-55% have metastasis when diagnosed (39). As corroborated, the average follow-up time of all patients in our study is only about one year. These factors might weaken the influence of their biological behaviors on patients’ suicide tendencies to some extent.

In the study of the influence of treatment on suicide risk, our research is the first to suggest a significant reduction in the risk of suicide in pancreatic patients who received chemotherapy or cancer-directed surgery. Compared with patients who did not receive the corresponding treatment, both chemotherapy and surgery were significantly associated with lower rates of suicide, with HRs of 0.456 (chemotherapy, 95% CI: 0.323-0.646, *P* < 0.001) and 0.553 (surgery, 95% CI: 0.342-0.895, *P* = 0.016), respectively. As indicated, the effect of surgery on suicide in patients with pancreatic cancer has been controversial in the previous study (12). In recent years, significant progress has been made in the treatment methods of pancreatic cancer (40, 41). The 5-year survival rate of patients with surgical resection and adjuvant therapy is as high as 30% (41). More than half of patients with borderline resectable tumors or even locally advanced unresectable pancreatic cancer have obtained surgical indications after neoadjuvant therapy (41). Current guidelines routinely recommend neoadjuvant therapy for all patients with borderline resectable pancreatic cancer in well physical condition, including nanoparticle albumin-bound paclitaxel plus gemcitabine and Combined with sequential chemoradiotherapy or modified FOLFIRINOX solution (30, 40). Predictably, as treatment approaches improve and indications for surgical resection expand, patients with pancreatic cancer would be better able to benefit from treatment, thus reducing their psychological burden and suicide rate. Screening and prevention of subsequent suicide should be enhanced for pancreatic cancer patients with adverse risk factors, such as unmarried, white, or male patients. Moreover, considering most pancreatic cancer patients committed suicide within one year of diagnosis in our cohort, it is reasonable to assume that for patients diagnosed with pancreatic cancer, timely cancer-directed treatment might help reduce the risk of subsequent suicide.

For suicide prevention strategies in pancreatic cancer patients, the first step should be establishing a strict identification process for patients with high-risk factors. Some studies recommended using questionnaires to diagnose and screen key psychological symptoms and early detect potential psychological problems in high-risk groups to give timely treatment intervention (42). Timely treatment for depression is also thought to have a significant effect on reducing suicide among cancer patients. Studies indicated that appropriate cancer treatment also reduces suicide rates (31). In addition, palliative care for patients with advanced pancreatic cancer is also feasible to improve the symptoms of patients and increase their comfort in life. In the social aspect, the support of family or friends and increased medical insurance coverage are also needed to a certain extent (12).

The following limitations remained in our study. Overall, suicide still accounted for a small percentage of deaths among pancreatic cancer patients (0.10%), and more detailed studies focusing on subgroups have not been available in the SEER database. Secondly, bias and confoundings are inevitable as a common shortcoming of retrospective studies. On the other hand, preventing the attempt to commit suicide might be more important in some ways. However, given the difficulty of conducting prospective studies, real-world studies of big data are still indispensable in suicide research, as highlighted (43). In addition, despite significant advances in treatment, pancreatic cancer patients still suffer a poor prognosis for various reasons, and the short mean follow-up time may interfere with studies of suicidal behavior. However, as confirmed in previous studies, there was a significant decline in suicide attempts over time after diagnosis, with most suicides in our study occurring within one year of diagnosis of pancreatic cancer. The study of suicide in the first year after diagnosis has been sufficient to illuminate critical issues.

## Conclusion

The 199,604 pancreatic cancer patients diagnosed between 2000 and 2018 had an overall suicide rate of 88.05 per 100,000 person-years and an SMR of 6.43 compared to the U.S. general population. Male, white, unmarried, and diagnosed with pancreatic adenocarcinoma patients were associated with a higher risk of suicide, while cancer-directed surgery and chemotherapy might be indicated protective factors. The screening and prevention process should be enhanced for pancreatic cancer patients with adverse risk factors. Moreover, it is reasonable to assume that timely cancer-directed treatment might help reduce the subsequent suicide risk of pancreatic cancer patients.

## Data availability statement

The datasets presented in this study can be found in online repositories. The names of the repository/repositories and accession number(s) can be found below: https://seer.cancer.gov/.

## Author contributions

Study concepts, ZWa, QM, and HH. Study design, ZWa, QM, and HH. Data acquisition, YM, JL, BY, and TY. Quality control of data and algorithms, QM, ZWu, and ZWa. Data analysis and interpretation, YM, JL, BY, and TY. Statistical analysis, YM, JL, and BY. Manuscript preparation, YM and HH. Manuscript editing, YM, JL, and TY. Manuscript review, ZWa, QM, HH, and ZWu. All authors contributed to the article and approved the submitted version.

## Funding

This work was supported by the National Natural Science Foundation of China (NSFC 81872008, 82072702), the National Key Research and Development Program of China (2019YFC1315900 and subproject 2019YFC1315902), the Science and Technology Innovation as a Whole Plan Projects of Shaanxi Province, China (No. 2016KJZDSF01-05-01), the Science and Technology Development of Shaanxi Province, China (No.2019SF-140), and Clinical Research Award of the First Affiliated Hospital of Xi'an Jiaotong University, China (No. XJTU1AF-CRF-2019- 005).

## Acknowledgments

The authors thank all colleagues who contributed to this effort.

## Conflict of interest

The authors declare that the research was conducted in the absence of any commercial or financial relationships that could be construed as a potential conflict of interest.

## Publisher’s note

All claims expressed in this article are solely those of the authors and do not necessarily represent those of their affiliated organizations, or those of the publisher, the editors and the reviewers. Any product that may be evaluated in this article, or claim that may be made by its manufacturer, is not guaranteed or endorsed by the publisher.

## References

[1] Zaorsky NG Zhang Y Tuanquin L Bluethmann SM Park HS Chinchilli VM. Suicide among cancer patients. Nat Commun (2019) 10:207. doi: 10.1038/s41467-018-08170-1 30643135 PMC6331593

[2] Garnett MF Curtin SC Stone DM. Suicide mortality in the united states, 2000–2020. NCHS data brief, no 433. Hyattsville, MD: National Center for Health Statistics (2022). doi: 10.15620/cdc:114217 35312475

[3] Misono S Weiss NS Fann JR Redman M Yueh B. Incidence of suicide in persons with cancer. J Clin Oncol (2008) 26:4731–8. doi: 10.1200/JCO.2007.13.8941 PMC2653137 18695257

[4] Klaassen Z Yaguchi G Terris MK. How can we decrease suicide risk in cases of genitourinary cancer? Future Oncol (2015) 11:2113–5. doi: 10.2217/fon.15.131 26235177

[5] Siegel RL Miller KD Fuchs HE Jemal A. Cancer statistics, 2022. CA Cancer J Clin (2022) 72:7–33. doi: 10.3322/caac.21708 35020204

[6] Fang CK Chang MC Chen PJ Lin CC Chen GS Lin J. A correlational study of suicidal ideation with psychological distress, depression, and demoralization in patients with cancer. Support Care Cancer (2014) 22:3165–74. doi: 10.1007/s00520-014-2290-4 PMC4218975 24935648

[7] Tanriverdi D Cuhadar D Ciftci S. Does the impairment of functional life increase the probability of suicide in cancer patients? Asian Pac J Cancer Prev (2014) 15:9549–53. doi: 10.7314/apjcp.2014.15.21.9549 25422254

[8] Fang F Fall K Mittleman MA Sparen P Ye W Adami HO. Suicide and cardiovascular death after a cancer diagnosis. N Engl J Med (2012) 366:1310–8. doi: 10.1056/NEJMoa1110307 22475594

[9] Spoletini I Gianni W Caltagirone C Madaio R Repetto L Spalletta G. Suicide and cancer: where do we go from here? Crit Rev Oncol Hematol (2011) 78:206–19. doi: 10.1016/j.critrevonc.2010.05.005 20605728

[10] Anguiano L Mayer DK Piven ML Rosenstein D. A literature review of suicide in cancer patients. Cancer Nurs (2012) 35:E14–26. doi: 10.1097/NCC.0b013e31822fc76c 21946906

[11] Henson KE Brock R Charnock J Wickramasinghe B Will O Pitman A. Risk of suicide after cancer diagnosis in England. JAMA Psychiat (2019) 76:51–60. doi: 10.1001/jamapsychiatry.2018.3181 PMC6583458 30476945

[12] Turaga KK Malafa MP Jacobsen PB Schell MJ Sarr MG. Suicide in patients with pancreatic cancer. Cancer-Am Cancer Soc (2011) 117:642–7. doi: 10.1002/cncr.25428 PMC4704452 20824626

[13] Jacobsson L Ottosson JO. Initial mental disorders in carcinoma of pancreas and stomach. Acta Psychiatr Scand Suppl (1971) 221:120–7. doi: 10.1111/j.1600-0447.1971.tb02144.x 5286323

[14] Fras I Litin EM Pearson JS. Comparison of psychiatric symptoms in carcinoma of the pancreas with those in some other intra-abdominal neoplasms. Am J Psychiatry (1967) 123:1553–62. doi: 10.1176/ajp.123.12.1553 4381627

[15] Cosci F Fava GA Sonino N. Mood and anxiety disorders as early manifestations of medical illness: a systematic review. Psychother Psychosom (2015) 84:22–9. doi: 10.1159/000367913 25547421

[16] Seoud T Syed A Carleton N Rossi C Kenner B Quershi H. Depression before and after a diagnosis of pancreatic cancer: Results from a national, population-based study. Pancreas (2020) 49:1117–22. doi: 10.1097/MPA.0000000000001635 PMC7450395 32833946

[17] Saad AM Gad MM Al-Husseini MJ AlKhayat MA Rachid A Alfaar AS. Suicidal death within a year of a cancer diagnosis: A population-based study. Cancer-Am Cancer Soc (2019) 125:972–9. doi: 10.1002/cncr.31876 30613943

[18] Xu J Murphy SL Kochanek KD Arias E. Deaths: Final data for 2019. In: National vital statistics reports, vol. vol 70. . Hyattsville, MD: National Center for Health Statistics (2021). doi: 10.15620/cdc:106058

[19] Gordon-Dseagu VL Devesa SS Goggins M Stolzenberg-Solomon R. Pancreatic cancer incidence trends: evidence from the surveillance, epidemiology and end results (SEER) population-based data. Int J Epidemiol (2018) 47:427–39. doi: 10.1093/ije/dyx232 PMC5913617 29149259

[20] Camp RL Dolled-Filhart M Rimm DL. X-Tile: a new bio-informatics tool for biomarker assessment and outcome-based cut-point optimization. Clin Cancer Res (2004) 10:7252–9. doi: 10.1158/1078-0432.CCR-04-0713 15534099

[21] Luo G Fan Z Gong Y Jin K Yang C Cheng H. Characteristics and outcomes of pancreatic cancer by histological subtypes. Pancreas (2019) 48:817–22. doi: 10.1097/MPA.0000000000001338 31210663

[22] Chen C Lin H Xu F Liu J Cai Q Yang F. Risk factors associated with suicide among esophageal carcinoma patients from 1975 to 2016. Sci Rep (2021) 11:18766. doi: 10.1038/s41598-021-98260-w 34548616 PMC8455550

[23] Chen C Jiang Y Yang F Cai Q Liu J Wu Y. Risk factors associated with suicide among hepatocellular carcinoma patients: A surveillance, epidemiology, and end results analysis. Eur J Surg Oncol (2021) 47:640–8. doi: 10.1016/j.ejso.2020.10.001 PMC7538389 33051117

[24] Ury HK Wiggins AD. Another shortcut method for calculating the confidence interval of a poisson variable (or of a standardized mortality ratio). Am J Epidemiol (1985) 122:197–8. doi: 10.1093/oxfordjournals.aje.a114083 4014198

[25] Bjorkenstam C Edberg A Ayoubi S Rosen M. Are cancer patients at higher suicide risk than the general population? Scand J Public Health (2005) 33:208–14. doi: 10.1080/14034940410019226 16040462

[26] Yousaf U Christensen ML Engholm G Storm HH. Suicides among Danish cancer patients 1971-1999. Br J Cancer (2005) 92:995–1000. doi: 10.1038/sj.bjc.6602424 15756279 PMC2361949

[27] Hem E Loge JH Haldorsen T Ekeberg O. Suicide risk in cancer patients from 1960 to 1999. J Clin Oncol (2004) 22:4209–16. doi: 10.1200/JCO.2004.02.052 15483032

[28] Franklin JC Ribeiro JD Fox KR Bentley KH Kleiman EM Huang X. Risk factors for suicidal thoughts and behaviors: A meta-analysis of 50 years of research. Psychol Bull (2017) 143:187–232. doi: 10.1037/bul0000084 27841450

[29] Boyd AD Riba M. Depression and pancreatic cancer. J Natl Compr Canc Netw (2007) 5:113–6. doi: 10.6004/jnccn.2007.0012 17239331

[30] Mizrahi JD Surana R Valle JW Shroff RT. Pancreatic cancer. Lancet (2020) 395:2008–20. doi: 10.1016/S0140-6736(20)30974-0 32593337

[31] Gage-Bouchard EA Pailler M Devine KA Flores T. Optimizing patient-centered psychosocial care to reduce suicide risk and enhance survivorship outcomes among cancer patients. J Natl Cancer Inst (2021) 113:1129–30. doi: 10.1093/jnci/djaa185 PMC8418430 33464289

[32] Sugawara A Kunieda E. Suicide in patients with gastric cancer: a population-based study. Jpn J Clin Oncol (2016) 46:850–5. doi: 10.1093/jjco/hyw075 27307574

[33] Suominen K Isometsa E Suokas J Haukka J Achte K Lonnqvist J. Completed suicide after a suicide attempt: a 37-year follow-up study. Am J Psychiatry (2004) 161:562–3. doi: 10.1176/appi.ajp.161.3.562 14992984

[34] Salk RH Hyde JS Abramson LY. Gender differences in depression in representative national samples: Meta-analyses of diagnoses and symptoms. Psychol Bull (2017) 143:783–822. doi: 10.1037/bul0000102 28447828 PMC5532074

[35] Zhou H Xian W Zhang Y Chen G Zhao S Chen X. Trends in incidence and associated risk factors of suicide mortality in patients with non-small cell lung cancer. Cancer Med (2018) 7:4146–55. doi: 10.1002/cam4.1656 PMC6089196 29971970

[36] Neeleman J Wessely S Lewis G. Suicide acceptability in African- and white americans: the role of religion. J Nerv Ment Dis (1998) 186:12–6. doi: 10.1097/00005053-199801000-00003 9457142

[37] Assari S Lankarani MM. Depressive symptoms are associated with more hopelessness among white than black older adults. Front Public Health (2016) 4:82. doi: 10.3389/fpubh.2016.00082 27200335 PMC4854870

[38] Hietanen P Lonnqvist J. Cancer and suicide. Ann Oncol (1991) 2:19–23. doi: 10.1093/oxfordjournals.annonc.a057816 2009232

[39] Park W Chawla A O’Reilly EM. Pancreatic cancer: A review. JAMA (2021) 326:851–62. doi: 10.1001/jama.2021.13027 PMC9363152 34547082

[40] Strobel O Neoptolemos J Jager D Buchler MW. Optimizing the outcomes of pancreatic cancer surgery. Nat Rev Clin Oncol (2019) 16:11–26. doi: 10.1038/s41571-018-0112-1 30341417

[41] Neoptolemos JP Kleeff J Michl P Costello E Greenhalf W Palmer DH. Therapeutic developments in pancreatic cancer: current and future perspectives. Nat Rev Gastroenterol Hepatol (2018) 15:333–48. doi: 10.1038/s41575-018-0005-x 29717230

[42] Ullman K. Reducing risk of suicide in cancer patients. J Natl Cancer Inst (2017) 109. doi: 10.1093/jnci/djx025 28800700

[43] Sun M Trinh QD. A surveillance, epidemiology and end results (SEER) database malfunction: perceptions, pitfalls and verities. Bju Int (2016) 117:551–2. doi: 10.1111/bju.13226 26190064

